# Incidence and extent of TDP-43 accumulation in aging human brain

**DOI:** 10.1186/s40478-015-0215-1

**Published:** 2015-06-20

**Authors:** Akiko Uchino, Masaki Takao, Hiroyuki Hatsuta, Hiroyuki Sumikura, Yuta Nakano, Akane Nogami, Yuko Saito, Tomio Arai, Kazutoshi Nishiyama, Shigeo Murayama

**Affiliations:** Department of Neuropathology (Brain Bank for Aging Research), Tokyo Metropolitan Geriatric Hospital & Institute of Gerontology, 35-2 Sakae-cho, Itabashi-ku, Tokyo, 173-0015 Japan; Department of Neurology, Kitasato University School of Medicine, Sagamihara-shi, Kanagawa, Japan; Department of Neurology, Saitama Medical University International Medical Center, Hidaka-shi, Saitama, Japan; Department of Laboratory Medicine, National Center Hospital for Neurology and Psychiatry, Kodaira-shi, Tokyo, Japan; Department of Pathology, Tokyo Metropolitan Geriatric Hospital & Institute of Gerontology, Itabashi-ku, Tokyo, Japan; Department of Neurology, Tokyo Metropolitan Geriastric Hospital & Institute of Gerontrogy, Itbashi-ku, Tokyo, Japan

**Keywords:** TDP-43, Aging, Hippocampus, Uncus, Amygdala

## Abstract

**Introduction:**

The transactivation response element DNA-binding protein 43 kDa (TDP-43) is a major component of the ubiquitin-positive and tau-negative inclusions in frontotemporal lobar degeneration and sporadic amyotrophic lateral sclerosis (ALS). TDP-43 may accumulate in cases of Alzheimer’s disease (AD), Lewy body disease (LBD), and argyrophilic grain disease (AGD). However, few studies have focused on the incidence and extent of TDP-43 deposition in aging.

**Results:**

We analyzed 286 consecutive autopsy brains neuropathologically. Of these, 136 brains with pathologically minimal senile changes were designated as control elderly brains (78.5 ± 9.7 y). For comparison, we selected 29 AD, 11 LBD, and 11 AGD patients from this series of autopsy brains. Sections of the hippocampus, amygdala, medulla oblongata, and lumbar spinal cord were immunostained with anti-phosphorylated TDP-43 antibody (PSer409/410). TDP-43 immunoreactive structures were classified into four types: dystrophic neurites (DNs), neuronal or glial cytoplasmic inclusions, and intranuclear inclusions. TDP-43 immunoreactive structures were observed in 55/136 control elderly (40.0 %), 21/29 AD (72.4 %), 8/11 LBD (72.7 %), and 6/11 AGD (54.5 %) brains. TDP-43 immunoreactive structures in control elderly brains were mostly DNs. These DNs were predominantly present in the uncus of the anterior hippocampus over age 65. The frequency of cases with DNs in the amygdala of control elderly brains was less than that of AD, LBD, and AGD brains. The mean age at death was significantly higher in cases with TDP-43 immunoreactive structures than cases without them.

**Conclusions:**

In conclusion, TDP-43 immunoreactive DNs may develop as a consequence of aging processes in the human brain. In particular, the uncus of the anterior hippocampus is an area highly susceptible to TDP-43 accumulation over age 65.

## Introduction

The transactivation response element DNA-binding protein 43 kDa (TDP-43) is a major component of the ubiquitin-positive and tau-negative inclusions in frontotemporal lobar degeneration (FTLD-TDP) and sporadic amyotrophic lateral sclerosis (ALS) [1–3]. However, the cytopathology and distribution pattern of TDP-43 immunoreactive deposits generally differ between FTLD-TDP and ALS [3, 4].

Previous studies have indicated that TDP-43 immunoreactive inclusion bodies can be detected in other neurodegenerative disorders as well, including Alzheimer’s disease (AD) [5–9], Lewy body disease (LBD) [6, 8, 10], and argyrophilic grain disease (AGD) [11]. Furthermore, several studies have focused on TDP-43 deposition in the absence of neurodegenerative disorders [10, 12–14]. One such study demonstrated that TDP-43 deposition increased with age and did not occur in individuals younger than 65 years [12]. Another study indicated that the incidence of TDP-43 deposition in the amygdala and hippocampus of cognitively normal elderly individuals was 36 % [14]. These previous studies suggest that TDP-43 deposition may be associated not only with specific neurodegenerative disorders but also with aging and specific anatomical areas. To further analyze the functional role of TDP-43, it will be important to understand the anatomical distribution of TDP-43 immunoreactive deposits in a large number of elderly individuals with neuropathologically minimal changes. The aim of this study was to clarify the incidence and distribution of TDP-43 deposition in control elderly brains by immunohistochemical analysis of a series of autopsied individuals. We also analyzed TDP-43 immunoreactive deposits in individuals with AD, LBD, and AGD for comparison.

## Materials and methods

### Brain samples

Tissue samples were collected at the Tokyo Metropolitan Geriatric Hospital, which provides community-based medical service to the elderly population. From February 2008 to July 2012, we obtained the 286 consecutive autopsy brains (165 men and 121 women) used for this study. Patient ages ranged from 43 to 104 years (mean ± SD, 82.0 ± 9.6 y). Clinical information was retrospectively obtained from the medical chart. The Mini-Mental State examination (MMSE) [15] was used for evaluation of cognitive function, and the clinical dementia scale (CDR) [16] was employed for the grading of cognitive decline as previously reported [17]. The brain samples used in this study were registered to the Brain Bank for Aging Research (BBAR) with the deceased’s relatives’ informed consent to carry out comprehensive research. The BBAR is approved by the ethics committee of the Tokyo Metropolitan Geriatric Hospital and Institute of Gerontology.

### Neuropathology and immunohistochemistry

The brains and spinal cords were examined according to routine protocols used in our laboratory [18]. Briefly, the cerebral and cerebellar hemispheres as well as the brainstem were dissected in the midsagittal plane at the time of autopsy. In each case, part of the brain was stored at −80 °C for further biochemical and molecular analyses. The brains and spinal cords were fixed in 20 % buffered formalin (WAKO, Osaka, Japan) for 7–13 days. After fixation in formalin, the cerebral hemispheres, brainstem, and cerebellum were dissected along the coronal, axial, and sagittal planes, respectively. Representative anatomical areas were embedded in paraffin, and 6-μm-thick sections were obtained to diagnose each case neuropathologically. Sections were stained with hematoxylin and eosin (H&E) and the Klüver-Barrera methods. Selected sections were further examined with modified methenamine and Gallyas–Braak silver staining for senile changes, Congo red for amyloid deposition, and Elastica Masson trichrome stain for vascular changes. Selected sections were immunostained with a Ventana BenchMark GX autostainer (Ventana Medical Systems, Tucson, AZ, USA) and an I-View Universal DAB Detection Kit (Roche, Basel, Switzerland) in accordance with the manufacturer’s instructions. The antibodies employed are shown in Table 1. Histologic sections were observed under a research microscope (Eclipse 90i; Nikon, Tokyo, Japan).

**Table 1:** Antibodies used for immunohistochemistry

Antibody	Type	Dilution	Source
Pser409/410	m	1:10000	A gift from M. Hasegawa, Japan
(phosphorylated TDP-43)			
anti-human amyloid β	m	1:50	IBL, Maebashi, Gunma, Japan
(11–28) (12B2)			
AT8	m	1:1000	Innogenetics, Ghent, Belgium
(phosphorylated tau)			
Psyn64	m	1:10000	A gift from T. Iwatsubo, Japan
(phosphorylated α-synuclein)			
ubiquitin	r	1:1000	Dako, Glostrup, Denmark

m, mouse monoclonal; r, rabbit polyclonal

### Case selection

**Fig. 1:**
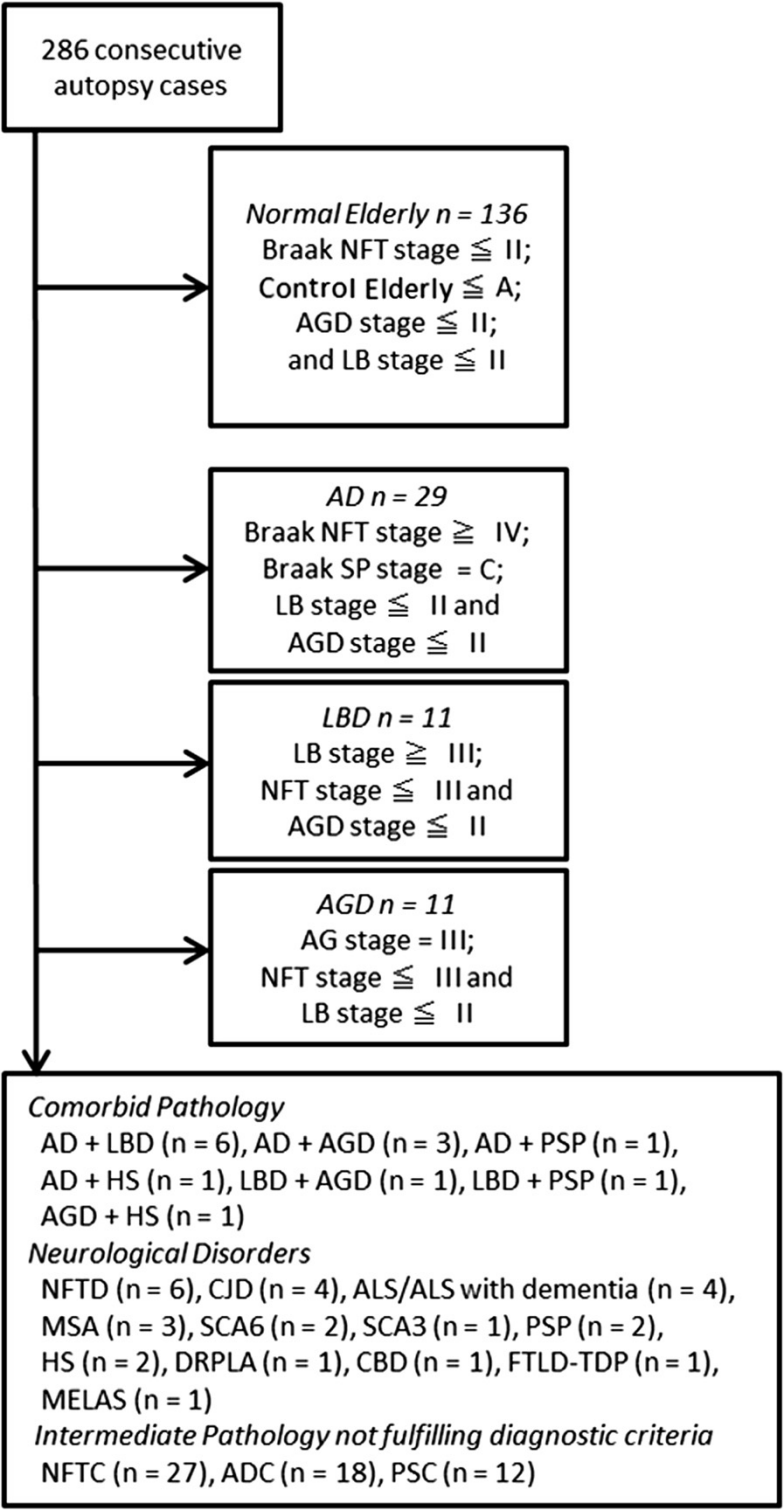
Case selection procedure. NFT, neurofibrillary tangle; SP, senile plaque; AGD, argyrophilic grain disease; LB, Lewy body; AD, Alzheimer’s disease; LBD, Lewy body disease (Parkinson’s disease and dementia with Lewy bodies); PSP, progressive supranuclear palsy; HS, hippocampal sclerosis; NFTD, NFT-predominant form of senile dementia; CJD, Creutzfeldt–Jakob disease; ALS, amyotrophic lateral sclerosis; MSA, multiple system atrophy; SCA, spinocerebellar ataxia; DRPLA, dentatorubral-pallidoluysian atrophy; CBD, corticobasal degeneration; FTLD, frontotemporal lobar degeneration; MELAS, mitochondrial encephalopathy with lactic acidosis and stroke-like episodes; NFTC, NFT-predominant change; ADC, AD-type change; PSC, plaque dominant senile change

The case selection procedure in this study is shown in Fig. 1. First, of the 286 consecutive autopsy cases, we selected cases that met all of the following conditions and defined them as control elderly cases that have minimal senile neuropathologic changes: Braak’s neurofibrillary tangle (NFT) stage II or less, senile plaque (SP) stage [19] A or less, AGD stage [17] II or less, and Lewy body stage [20] II or less. For comparison, we selected cases with AGD stage III as AGD and those with Lewy body stage above III as LBD. We also defined AD according to our brain bank definition (Braak NFT stage above IV and Braak’s SP stage C) [21].

As to the cases with comorbid pathologies and other neurological disorders, we also provided the results of TDP-43 pathology. However, they were not used for comparison with control elderly cases because they were limited in number: AD plus LBD (n = 6), AD plus AGD (n = 3), AD plus progressive supranuclear palsy (PSP) [22] (n = 1), AD plus hippocampal sclerosis (HS) (n = 1), LBD plus AGD (n = 1), LBD plus PSP (n = 1), AGD plus HS (n = 1), senile dementia with tangles (NFTD) (n = 6) [23], Creutzfeldt–Jakob disease (CJD) (n = 4), ALS/ALS with dementia (n = 4), multiple system atrophy (MSA) (n = 3), spinocerebellar ataxia type 6 (SCA6) (n = 2), spinocerebellar ataxia type 3 (SCA3) (n = 1), PSP (n = 2), HS (n = 2), dentatorubral–pallidoluysian atrophy (DRPLA) (n = 1), corticobasal degeneration (CBD) (n = 1), FTLD-TDP (n = 1), and mitochondrial encephalopathy with lactic acidosis and stroke-like episodes (MELAS) (n = 1). Of the remaining cases, we identified the followings as intermediate pathologies that did not fulfill diagnostic criteria: Braak NFT stage above III and Braak’s SP stage A as neurofibrillary tangle-predominant change (NFTC, n = 27), Braak NFT stage above III and Braak’s SP stage above B as AD type change (ADC, n = 18), and Braak NFT stage II or less and Braak’s SP stage above B as plaque dominant senile change (PSC, n = 12).

### Evaluation of severity and distribution of TDP-43 immunoreactivity

**Fig. 2:**
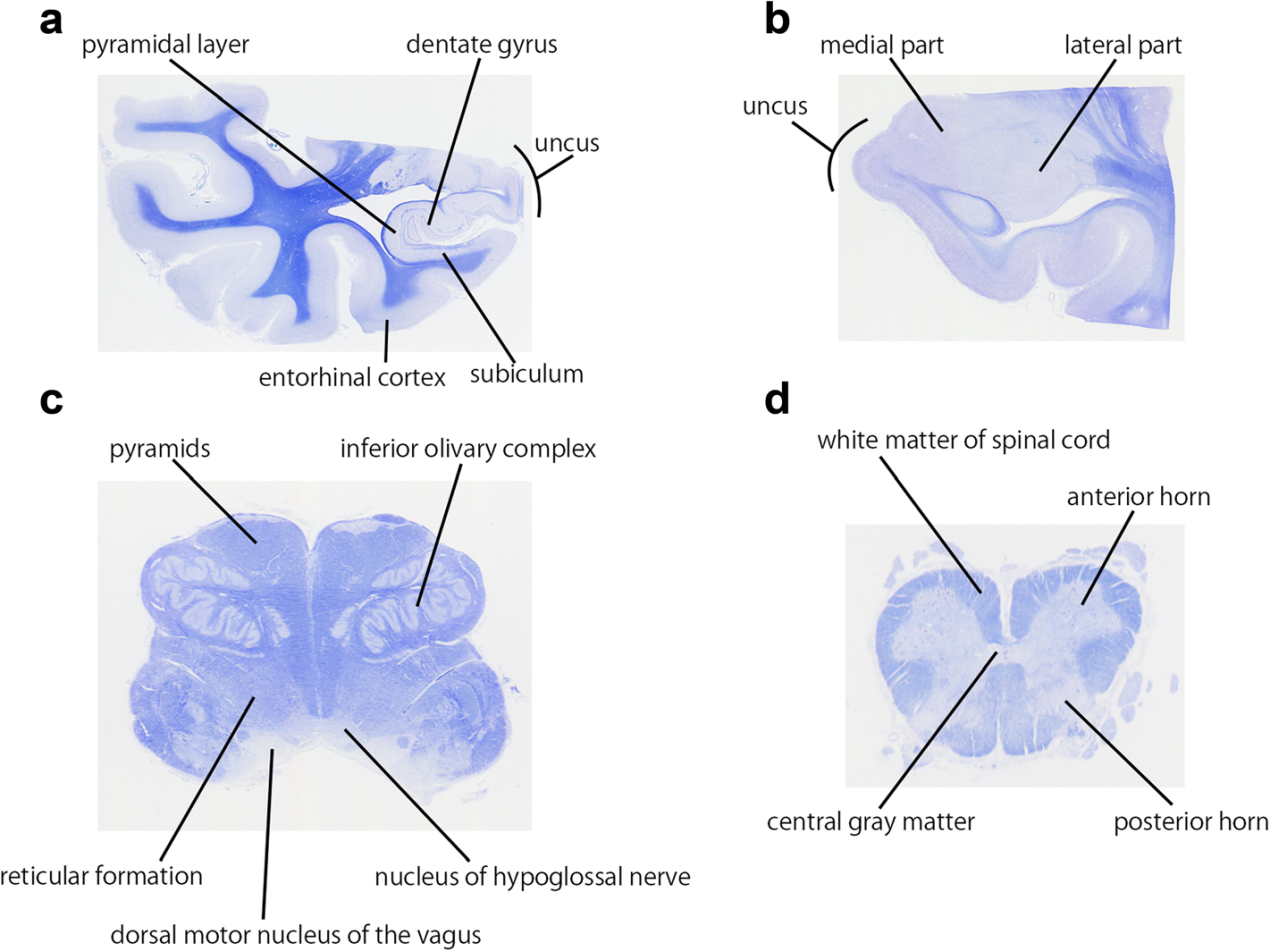
Evaluated anatomical regions in this study. **a** anterior hippocampus; **b** amygdala; **c** medulla oblongata; **d** lumbar spinal cord. Each anatomical region was identified using the neuroanatomy atlas [24]. The uncus was defined and identified using the human hippocampus, third edition [25]

The presence and severity of TDP-43 immunoreactive structures were assessed in four brain sections including the anterior hippocampus, amygdala, medulla oblongata, and lumbar spinal cord. In fact, the anterior hippocampus is key anatomical structures of FTLD-TDP. In addition, TDP-43 immunoreactive deposits have been well analyzed in AD, DLB and AGD in the limbic regions [5, 6, 8–11]. The medulla oblongata and spinal cord are important areas in association with ALS with TDP-43 [4]. Therefore, we considered that those four areas are appropriate for the study about TDP-43 pathology in control elderly brains. We assessed TDP-43 immunoreactive structures in each anatomical region shown in Table 2 and Fig. 2 under a 10× objective. Each anatomical region was identified using the neuroanatomy atlas [24]. The uncus was identified and analyzed at the level of amygdala and anterior hippocampus [25]. Neuronal cytoplasmic inclusions (NCIs), glial cytoplasmic inclusions (GCIs), and neuronal intranuclear inclusions (NIIs) immunoreactive for TDP-43 were quantitatively analyzed and scored as 0 to 3 depending on the total number of TDP-43 immunoreactive NCIs, GCIs, or NIIs: 0, none; 1, 1–3; 2, 4–9; and 3, ≥10. TDP-43 immunoreactive dystrophic neurites (DNs) were semi-quantitatively scored as 0 to 3: 0, absent; 1, sparse; 2, moderate; and 3, severe.

**Table 2:** Brain regions analyzed in this study

area	regions
Anterior hippocampus	dentate gyrus
	uncus
	pyramidal layer
	subiculum
	entorhinal cortex
Amygdala	cortical amygdaloid nucleus as medial part
	lateral part including basolateral, lateral amygdaloid nucleus
	uncus
Medulla oblongata	dorsal motor nucleus of the vagus
	nucleus of hypoglossal nerve
	inferior olivary complex
	reticular formation
	pyramids
Lumbar spinal cord	anterior horn
	posterior horn
	white matter of spinal cord
	central gray matter

### Statistical analysis

The Mann–Whitney *U* test was used to compare the age at death and brain weight between TDP-43–positive and TDP-43–negative groups. Fisher’s exact test was used to compare the male-to-female ratio and number of TDP-43 structures. A *p* value of less than 0.05 was considered statistically significant.

## Results

The demographics of the cases in this study are shown in Table 3. We selected 136 cases as control elderly (mean age at death ± 1 SD, 78.5 ± 9.7 y). For comparison, we selected 29 AD, 11 LBD, and 11 AGD cases from the series of autopsied individuals. Compared with the control elderly group, the mean age at death was higher for AD, LBD, and AGD groups (Table 3).

**Table 3:** Demographics of cases included in this study

	Control elderly	AD	LBD	AGD
Number (case)	136	29	11	11
Male, Female	95, 41	12, 17	9, 2	4, 7
Mean age at death	78.5 ± 9.7*	86.0 ± 6.2	84.0 ± 5.6	86.6 ± 6.3
(years, mean ± SD)				
M ean brain weight	1257 ± 129	1168 ± 185	1208 ± 77.4	1159 ± 129
(grams, mean ± SD)				

AD, Alzheimer’s disease; LBD, Lewy body disease; AGD, Argyrophilic grain disease; SD, standard deviation

*, *p* < 0.05 compared with AD, LBD and AGD

### Distribution of TDP-43 pathology in control elderly brains

**Fig. 3:**
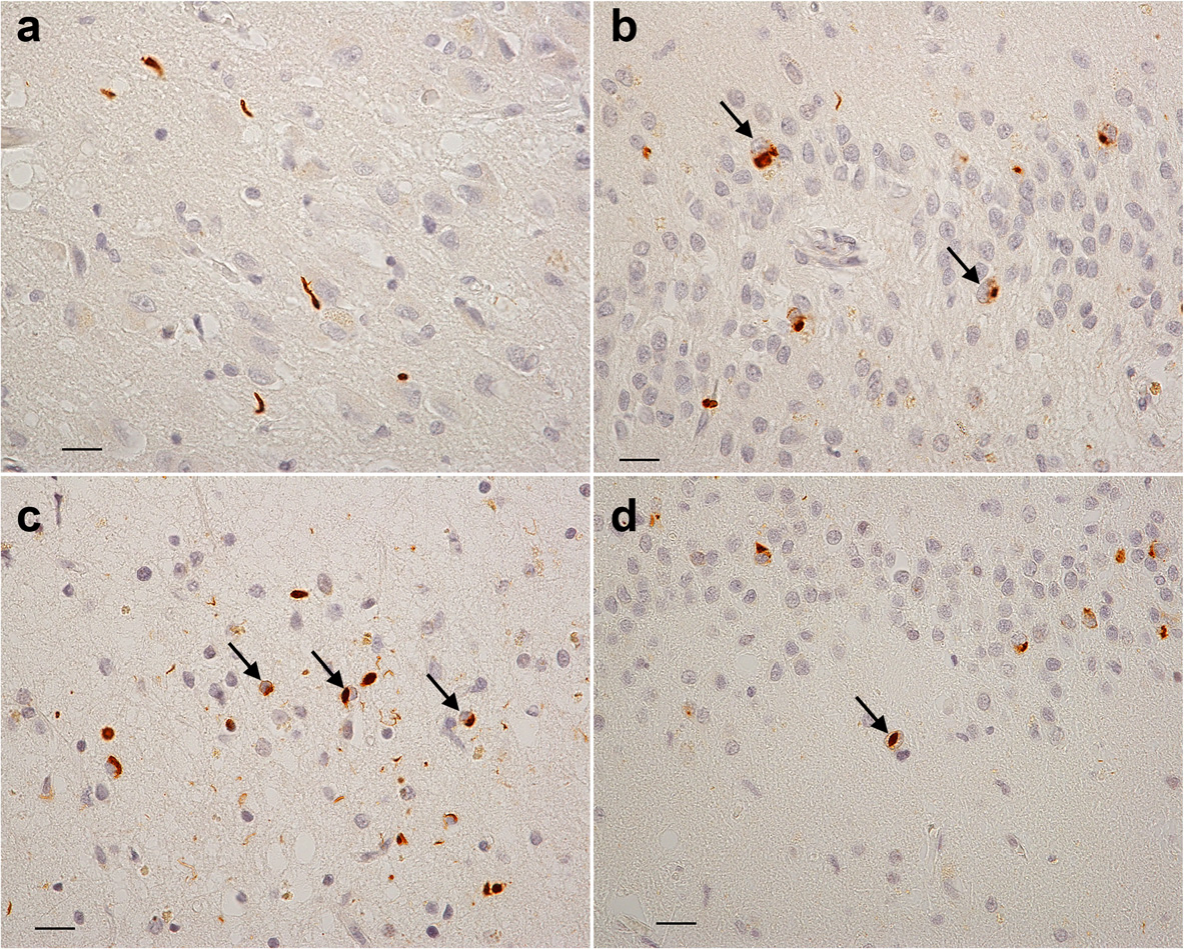
TDP-43 immunoreactive structures in representative cases. **a** Dystrophic neurites in the uncus of the anterior hippocampus in the control elderly brain. **b** Neuronal cytoplasmic inclusions (*arrows*) in the dentate gyrus in Alzheimer’s disease (AD). **c** Glial cytoplasmic inclusions (*arrows*) in the entorhinal cortex in AD. **d** Neuronal intranuclear inclusion (*arrow*) in the dentate gyrus in AD. Scale bar = 20 μm

TDP-43 immunoreactive structures were found in 55/136 (40.0 %) cases (Table 4). Most of these structures were DNs (53/136 cases, 39.0 %; Table 5); they were predominantly detected at the uncus of the anterior hippocampus (29/136 cases, 21.3 %; Table 5, Fig. 3a). In rare instances, DNs were present in other limbic areas such as the uncus of the amygdala (5/134 cases, 3.7 %; two cases of amygdala were unavailable, Table 5). In control elderly brains, TDP-43 immunoreactive GCIs and NCIs were rare. In fact, GCIs were observed only in a 94-year-old woman’s entorhinal cortex and NCIs only in a 98-year-old woman’s subiculum and pyramidal layer (Table 5). No NIIs were present in control elderly brains. TDP-43 immunoreactive DNs were also found in the inferior olivary complex (14/136 cases, 10.3 %), anterior horn (7/136 cases, 5.1 %), and white matter of the lumbar spinal cord (11/136 cases, 8.1 %), as shown in Table 5. No NCIs or GCIs were found in the medulla oblongata or lumbar spinal cord.

**Table 4:** Presence of TDP-43 pathology

	Control elderly			AD			LBD			AGD		
	positive	negative	*p* value	positive	negative	p value	positive	negative	*p* value	positive	negative	*p* value
Cases (%)	55 (40 %)	81 (60 %)		21 (72.4 %)	8 (27.6 %)		8 (72.7 %)	3 (27.3 %)		6 (54.5 %)	5 (45.5 %)	
Male, Female	41, 14	54, 27	N.S.	9, 12	3, 5	N.S.	6, 2	3, 0	N.S.	2, 4	2, 3	N.S.
Mean age at death	80.6 ± 9.3	77.1 ± 9.7	0.038	86.2 ± 6.8	85.5 ± 4.5	N.S.	85.1 ± 4.6	81.0 ± 6.7	N.S.	90.3 ± 4.8	82.2 ± 4.7	0.028
(years, mean ± SD)												
Mean brain weight	1254 ± 115	1259 ± 37	N.S.	1168 ± 208	1169 ± 103	N.S.	1217 ± 57.2	1184 ± 111	N.S.	1149 ± 105	1171 ± 153	N.S.
(grams, mean ± SD)												

AD, Alzheimer’s disease; LBD, Lewy body disease; AGD, Argyrophilic grain disease; SD, standard deviation; N.S., not significant

**Table 5:** TDP-43 distribution in this study

		Control elderly				AD				LBD				AGD			
		n = 136				n = 29				n = 11				n = 11			
		score				score				score				score			
		0	1	2	3	0	1	2	3	0	1	2	3	0	1	2	3
Dentate gyrus	DN	135	0	0	0	20	4	0	1	11	0	0	0	10	0	0	0
	GCI	135	0	0	0	20	1	4	0	11	0	0	0	10	0	0	0
	NCI	135	0	0	0	18	2	2	3	10	1	0	0	10	0	0	0
	NII	135	0	0	0	22	3	0	0	11	0	0	0	10	0	0	0
Uncus (Hippocampus)	DN	107	13	12	4	17	4	4	3	6	3	2	0	6	4	1	0
	GCI	136	0	0	0	21	0	4	3	10	1	0	0	11	0	0	0
	NCI	136	0	0	0	17	2	3	6	11	0	0	0	10	0	1	0
	NII	136	0	0	0	27	1	0	0	11	0	0	0	11	0	0	0
Pyramidal layer	DN	136	0	0	0	21	2	3	2	11	0	0	0	11	0	0	0
	GCI	136	0	0	0	18	2	5	3	11	0	0	0	11	0	0	0
	NCI	135	1	0	0	16	3	6	3	10	1	0	0	11	0	0	0
	NII	136	0	0	0	27	1	0	0	11	0	0	0	11	0	0	0
Subiculum	DN	133	2	1	0	19	5	2	2	9	1	1	0	11	0	0	0
	GCI	136	0	0	0	16	4	3	5	11	0	0	0	11	0	0	0
	NCI	135	1	0	0	15	3	5	5	11	0	0	0	11	0	0	0
	NII	136	0	0	0	27	1	0	0	11	0	0	0	11	0	0	0
Entorhinal cortex	DN	135	0	0	0	17	5	1	5	10	1	0	0	11	0	0	0
	GCI	134	1	0	0	17	2	4	5	10	0	1	0	11	0	0	0
	NCI	135	0	0	0	19	2	3	4	10	0	1	0	11	0	0	0
	NII	135	0	0	0	24	4	0	0	10	1	0	0	11	0	0	0
Amygdala Medial part	DN	130	4	0	0	18	4	4	3	10	1	0	0	9	0	1	0
	GCI	134	0	0	0	17	3	7	2	10	0	1	0	10	0	0	0
	NCI	134	0	0	0	18	2	3	6	10	0	1	0	9	1	0	0
	NII	134	0	0	0	28	1	0	0	11	0	0	0	10	0	0	0
Amygdala Lateral part	DN	133	0	0	1	19	7	0	3	10	1	0	0	9	0	1	0
	GCI	134	0	0	0	18	4	5	2	10	0	1	0	9	0	1	0
	NCI	134	0	0	0	18	0	5	6	10	0	1	0	9	0	1	0
	NII	134	0	0	0	28	1	0	0	11	0	0	0	10	0	0	0
Uncus (Amygdala)	DN	129	3	2	0	15	8	3	3	8	3	0	0	7	2	1	0
	GCI	134	0	0	0	16	6	7	0	9	2	0	0	10	0	0	0
	NCI	134	0	0	0	16	6	3	4	10	1	0	0	9	1	0	0
	NII	134	0	0	0	29	0	0	0	11	0	0	0	10	0	0	0
Dorsal motor nucleus of the vagus	DN	136	0	0	0	27	2	0	0	11	0	0	0	11	0	0	0
	GCI	136	0	0	0	29	0	0	0	11	0	0	0	11	0	0	0
	NCI	136	0	0	0	29	0	0	0	11	0	0	0	11	0	0	0
	NII	136	0	0	0	29	0	0	0	11	0	0	0	11	0	0	0
Nucleus of hypoglossal nerve	DN	136	0	0	0	29	0	0	0	11	0	0	0	11	0	0	0
	GCI	136	0	0	0	29	0	0	0	11	0	0	0	11	0	0	0
	NCI	136	0	0	0	29	0	0	0	11	0	0	0	11	0	0	0
	NII	136	0	0	0	29	0	0	0	11	0	0	0	11	0	0	0
Inferior olivary complex	DN	122	11	3	0	25	2	2	0	7	4	0	0	9	2	0	0
	GCI	136	0	0	0	29	0	0	0	11	0	0	0	11	0	0	0
	NCI	136	0	0	0	29	0	0	0	11	0	0	0	11	0	0	0
	NII	136	0	0	0	29	0	0	0	11	0	0	0	11	0	0	0
Reticular formation	DN	132	2	2	0	28	1	0	0	11	0	0	0	11	0	0	0
	GCI	136	0	0	0	29	0	0	0	11	0	0	0	11	0	0	0
	NCI	136	0	0	0	29	0	0	0	11	0	0	0	11	0	0	0
	NII	136	0	0	0	29	0	0	0	11	0	0	0	11	0	0	0
Pyramids	DN	135	1	0	0	29	0	0	0	11	0	0	0	11	0	0	0
	GCI	136	0	0	0	29	0	0	0	11	0	0	0	11	0	0	0
	NCI	136	0	0	0	29	0	0	0	11	0	0	0	11	0	0	0
	NII	136	0	0	0	29	0	0	0	11	0	0	0	11	0	0	0
Anterior horn	DN	129	7	0	0	28	1	0	0	11	0	0	0	11	0	0	0
	GCI	136	0	0	0	29	0	0	0	11	0	0	0	11	0	0	0
	NCI	136	0	0	0	29	0	0	0	11	0	0	0	11	0	0	0
	NII	136	0	0	0	29	0	0	0	11	0	0	0	11	0	0	0
Posterior horn	DN	134	2	0	0	29	0	0	0	11	0	0	0	11	0	0	0
	GCI	136	0	0	0	29	0	0	0	11	0	0	0	11	0	0	0
	NCI	136	0	0	0	29	0	0	0	11	0	0	0	11	0	0	0
	NII	136	0	0	0	29	0	0	0	11	0	0	0	11	0	0	0
White matter	DN	125	11	0	0	27	2	0	0	11	0	0	0	10	1	0	0
Central gray matter	DN	133	3	0	0	29	0	0	0	11	0	0	0	11	0	0	0
	GC	136	0	0	0	29	0	0	0	11	0	0	0	11	0	0	0
	NC	136	0	0	0	29	0	0	0	11	0	0	0	11	0	0	0
	NN	136	0	0	0	29	0	0	0	11	0	0	0	11	0	0	0

AD, Alzheimer’s disease; LBD, Lewy body disease; AGD, Argyrophilic grain disease; GCI, glial cytoplasmic inclusion; NCI, neuronal cytoplasmic inclusion; NII, neuronal intranuclear inclusion. The dentate gyrus was unavailable in one case of control elderly, 3 cases of AD, and one case of AGD. The amygdala was unavailable in two cases of control elderly and one case of AGD. The entorhinal cortex was unavailable in one case of control elderly. The anterior hippocampus was unavailable in one case of AD

### Distribution of TDP-43 pathology in AD, LBD, and AGD brains

#### AD brains

TDP-43–positive structures were found in 21/29 (72.2 %) AD brains and were widely distributed in the amygdala and hippocampus (Tables 4, 5). These structures were composed of NCIs (17/29 cases, 58.6 %) and GCIs (18/29 cases, 62.1 %) as well as DNs (19/29 cases, 65.5 %) (Fig. 3b, c). DNs were observed in the uncus of the anterior hippocampus (11/28 cases, 39.3 %; in one case, the anterior hippocampus was unavailable) as well as in the amygdala (14/29 cases, 48.3 %). In 7/29 cases (24.1 %), TDP-43 immunoreactive NIIs were also present in the amygdala and hippocampus (Fig. 3d). To a lesser degree, TDP-43–positive DNs were found in the medulla oblongata (5/29 cases; 17.2 %) and lumbar spinal cord (3/29 cases; 10.3 %). There were neither NCIs nor GCIs in any case of AD (Table 5).

#### LBD brains

TDP-43 immunoreactive structures were found in 8/11 (72.7 %) LBD brains and predominantly observed in the uncus of the anterior hippocampus and amygdala (Tables 4, 5). To a lesser degree, they were also found in the amygdaloid nuclei, entorhinal cortex, subiculum, and pyramidal cells of the hippocampus. TDP-43 immunoreactive structures mainly took the form of DNs (8/11 cases, 72.7 %), and occasionally of NCIs or GCIs in the hippocampus and amygdala (2/11 cases, 18.2 %). DNs were observed in the uncus of the anterior hippocampus (5/11 cases, 45.5 %) and in the amygdala (3/11 cases, 27.3 %). TDP-43 immunoreactive NIIs were found in the entorhinal cortex of only one case. In the medulla oblongata, TDP-43 immunoreactive DNs were present in the inferior olivary complex (4/11 cases, 36.4 %; Table 5). There were no DNs in the spinal cord. No NCIs or GCIs were found in any case of LBD.

#### AGD brains

TDP-43 immunoreactive structures were found in 6/11 (54.5 %) AGD brains and observed in the uncus and amygdaloid nuclei (Tables 4, 5). These structures were DNs (6/11 cases, 54.5 %). TDP-43 immunoreactive NCIs or GCIs were also found in the amygdaloid nuclei (3/11 cases, 27.3 %). DNs were observed in the uncus of the anterior hippocampus (5/11 cases, 45.5 %) and amygdala (3/10 cases, 33.3 %; one case was unavailable). No TDP-43–positive NIIs were found in these brains. In the medulla oblongata, we found TDP-43 immunoreactive DNs in the inferior olivary complex in two cases (2/11 cases, 18.2 %; Table 5). In the lumbar spinal cord, we found TDP-43 immunoreactive DNs in the white matter in an 85-year-old male. Neither NCIs nor GCIs were found in any of the AGD brains.

### Age and frequencies of DNs in four groups

The mean age at death was significantly higher in cases with TDP-43 immunoreactive structures than in those without TDP-43 immunoreactive structures in control elderly and AGD (Table 4). However, there were no significant differences in the mean age at death in AD and LBD (Table 4). Neither the brain weight nor sex ratio differed significantly among the four groups.

**Fig. 4:**
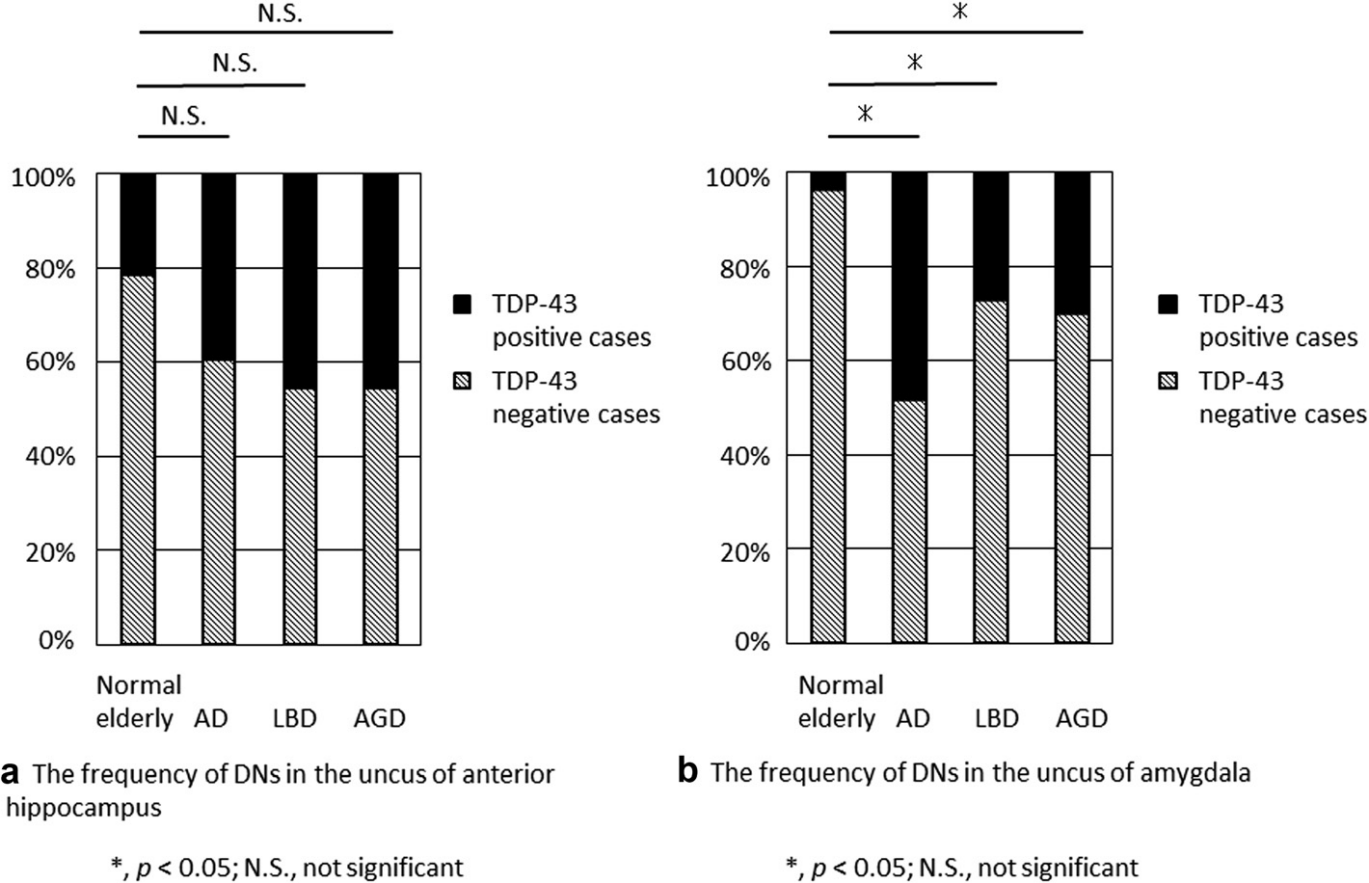
Incidence of TDP-43 immunoreactive dystrophic neurites (DNs) in the uncus of the anterior hippocampus and amygdala. **a** There were no significant differences in the number of cases with DNs in the uncus of the anterior hippocampus between control elderly brains and brains with Alzheimer’s disease (AD), Lewy body disease (LBD), and argyrophilic grain disease (AGD). **b** The incidence of DNs in the amygdala was significantly higher in the AD, LBD, and AGD brains than in control elderly brains

Because DNs were the predominant form of TDP-43 immunoreactive structure and observed mostly in the uncus in control elderly, we analyzed the frequencies of DNs in the uncus of the anterior hippocampus and amygdala in comparison with those of AD, LBD, and AGD. There were no significant differences in the frequency of cases with DNs in the uncus of the anterior hippocampus between control elderly and disease controls (Fig. 4a). In contrast, the frequency of cases with DNs observed in the uncus of the amygdala was higher in AD, LBD, and AGD compared with control elderly (Fig. 4b).

**Fig. 5:**
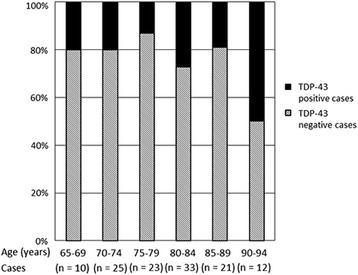
Correlation between age and incidence of TDP-43 immunoreactive structures in the uncus of the anterior hippocampus in control elderly brains. The incidence of TDP-43 positive brains ranged between 13 % and 27 % between ages 65 and 89 years. Although there were no significant differences, the incidence of TDP-43–positive brains increased to 50 % at ages 90 to 94 years

In addition, we focused on the association between the percentage of cases carrying DNs in the uncus of the anterior hippocampus and aging in control elderly. DNs were observed from age 65 to 94. In these individuals, there was no statistical association between the rate of DNs and aging (Fig. 5). However, the rate of DNs may increase beyond 90 years of age (Fig. 5).

### The relationship between TDP-43 immunoreactivity and cognitive function, cerebrovascular disease in control elderly brains

In control elderly brains, the rates of cases with cognitive impairment (CDR ≥ 0.5) among cases with and without TDP-43 immunoreactive structures were 38.3 % (18/47 cases, CDR was unavailable in 8 cases) and 34.3 % (28/70 cases, CDR was unavailable in 11 cases), respectively.

In addition, we examined the influence of cerebrovascular pathology. The rates of cases with cerebrovascular pathology among cases with and without TDP-43 immunoreactive structures were 61.2 % (34/55 cases) and 67.9 % (55/81 cases), respectively. There were no significant differences between cases with and without TDP-43 immunoreacrive structures.

### TDP-43 pathology in comorbid pathologies, other neurological disorders and intermediate pathologies.

TDP-43 immunoreactive structures were also found in the following cases: AD plus LBD (5/6 cases, 83.3 %); AD plus AGD (3/3 cases, 100 %); AD plus PSP (1/1 case, 100 %); AD plus HS (1/1 case, 100 %); LBD plus AGD (1/1 case, 100 %); LBD plus PSP (0/1 case, 0 %); AGD plus HS (1/1 case, 100 %); NFTD (3/6 cases, 50 %); CJD (0/4 cases, 0 %); ALS/ALS with dementia (4/4 cases, 100 %); MSA (2/3 cases, 66.7 %); SCA6 (2/2 cases, 100 %); SCA3 (0/1 case, 0 %); PSP (2/2 cases, 100 %); HS (2/2 cases, 100 %); DRPLA (0/1 case, 0 %); CBD (1/1 case, 100 %); FTLD-TDP (1/1 case, 100 %); MELAS (0/1 case, 0 %); NFTC (13/27 cases, 48.1 %); ADC (9/18 cases, 50 %); and PSC (6/12 cases, 50 %).

## Discussion

Our study is the first neuropathologic analysis that has clarified the anatomical distribution of TDP-43 immunoreactive structures in elderly brains with minimal senile neuropathologic changes in large numbers. We revealed the following findings. First, TDP-43 immunoreactive structures were present in 40 % of control elderly. Second, these structures were predominantly observed at the uncus of the anterior hippocampus as DNs over age 65. Third, the number of cases with DNs in the amygdala of control elderly was less than that of AD, LBD, or AGD. Fourth, no NCIs or GCIs were found in the medulla oblongata and lumbar spinal cord in all four groups. Finally, the mean age at death was significantly higher in cases with TDP-43 immunoreactive structures than those without such structures.

A few studies have assessed the frequency of TDP-43 immunoreactive structures in normal brain. Wilson *et al.* reported that 2/63 (3 %) were positive for TDP-43 in neurologically normal individuals ranging from 23 to 94 years old at death [13]. Another study of 110 cognitively normal elderly brains (mean age at death 86 ± 6.0) revealed that 40 cases (36.4 %) showed TDP-43 immunoreactivity in the limbic area [14]. In yet another study, 12 of 60 brains (20 %) without severe mental illness (mean age; 68) were reported as TDP-43 immunopositive in the amygdala or hippocampus [12]. The wide variation in the rate of TDP-43 immunoreactivity may depend on the age distribution of the sample population and the definition of a normal brain. In fact, the definition of a normal brain was based on the clinical information in previous studies. Because we defined the control elderly brain by using a neuropathologic staging system in association with neurodegenerative disorders and aging, the present study could control the effects of other protein deposits, including tau and α-synuclein, on TDP-43.

The distribution of TDP-43 immunoreactive structures in control elderly was different from those in AD. The structures in AD were widely present in the amygdala and hippocampus. Furthermore, in most cases, the TDP-43 immunoreactive structures in AD took the form of NCIs/GCIs, whereas only two cases of control elderly had GCIs or NCIs. These findings suggest that the pathological mechanism of TDP-43 accumulation in control elderly is different from that in AD. Although the amygdala was considered to be the first affected region of TDP-43 deposition in AD [26], TDP-43 immunoreactive structures in the amygdala may be non-specific. In this study, the frequency of cases with TDP-43 immunoreactive DNs observed in the uncus of the amygdala was significantly higher in AD than that in control elderly. This finding confirms that the sequential TDP-43 progression begins in the amygdala in AD. Interestingly, two control elderly cases over 90 showed NCIs or GCIs in the hippocampus and parahippocampus. Therefore, a portion of the TDP-43 immunoreactive NCIs and GCIs may be associated with aging. In addition, there is a possibility that these cases had neurodegenerative disorders at the earliest stages. Further cases need to be investigated to confirm these possibilities.

Previous studies that used a small number of cases also reported that TDP-43 immunoreactive structures were frequently observed in the amygdala in AD, LBD, and AGD [5, 6, 8–11]. In the present study, TDP-43 immunoreactive DNs in the amygdala of control elderly brains were less numerous than in that of AD, LBD or AGD brains. Therefore, TDP-43 immunoreactive deposits in the amygdala are associated with deposits of proteins such as tau and α-synuclein, which are related to other neurodegenerative disorders. In contrast, the number of cases with TDP-43 immunoreactive DNs in the uncus of the anterior hippocampus of control elderly showed no significant differences compared with AD, LBD, and AGD. Importantly, these DNs were present from age 65 to 94 years in our study. In particular, the proportion of TDP-43–positive cases was 13 % to 27 % in the age range of 65–89 years and 50 % in the age range of 90–94 years (Fig. 5). These findings suggest that TDP-43 accumulation in the uncus of the anterior hippocampus in the form of DNs is predominantly associated with physiological aging and not with neurodegenerative disorders.

We found TDP-43 immunoreactive DNs in the medulla oblongata and the spinal cord in control elderly brains and there were no NCIs or GCIs in all groups. These structures were predominantly located in the inferior olivary nuclei and the white matter of the spinal cord. The cases of AD, LBD, and AGD brains also showed TDP-43 immunoreactive DNs in those anatomical regions. We believe that the TDP-43 immunoreactive DNs in those specific anatomical regions may be associated with human physiological aging. In fact, the distribution pattern of TDP-43 deposits in this study clearly differs from that in ALS and FTLD-TDP brains that show many NCIs [4, 27].

Our study has some limitations. Because many aging brains have comorbid pathologies, the numbers of pure AD, LBD, and AGD brains in this study were relatively small. In addition, a part of TDP-43 accumulation of in AD, LBD, and AGD brains may be influenced by the aging process. In fact, for AD, LBD, and AGD, the mean age of death was significantly higher than that for control elderly.

Although TDP-43 has been associated with memory loss and medial temporal atrophy in AD [28], the role of TDP-43 accumulation in control elderly brains remains unresolved. The present study, we could not find the relationship between cognitive function and TDP-43 pathology in control elderly cases. Since the present study has not focused on neuropsychological examination, further studies in large numbers are needed to determine whether TDP-43 pathology might lead to subtle changes in cognitive function.

## Conclusions

In summary, our study clearly revealed that TDP-43 immunoreactive structures may develop as a consequence of aging processes in the human brain. Most of them were DNs not NCIs and GCIs, and were observed in the uncus of anterior hippocampus. Additional studies may be important to understand the role of TDP-43 accumulation in normal human aging. Besides those control elderly cases, we believe that the cases without TDP-43 immunoreactive deposits are also substantial for analysis of pathomechanism of TDP-43 accumulation.

## Availability of supporting data

The data sets supporting the results of this article are included within the article and its additional files.
